# Immunomodulatory Properties of Sweet Whey-Derived Peptides in THP-1 Macrophages

**DOI:** 10.3390/molecules30061261

**Published:** 2025-03-11

**Authors:** Eleni Dalaka, Georgios C. Stefos, Ioannis Politis, Georgios Theodorou

**Affiliations:** Laboratory of Animal Breeding and Husbandry, Department of Animal Science, Agricultural University of Athens, 11855 Athens, Greece; gstefos@aua.gr (G.C.S.); i.politis@aua.gr (I.P.)

**Keywords:** INFOGEST, cheese whey, inflammation, qPCR

## Abstract

Sweet whey (SW), a by-product of cheese production, has potential immunomodulatory properties that could be beneficial in preventing inflammation-related diseases. This study investigated the effects of SW derived from bovine, caprine, ovine, or an ovine/caprine mixture of milk on inflammation-related gene expression in THP-1-derived macrophages, both with and without LPS stimulation. Cells were treated with SW-D-P3 (a fraction smaller than 3 kDa produced by in vitro digestion), and the expression of inflammation-related genes was assessed using quantitative PCR. Results showed that the expression of *TLR2* and *ICAM1* was attenuated in non-LPS-stimulated macrophages treated with SW-D-P3, regardless of animal origin. Moreover, the expression of *TLR4*, *IL1B,* and *IL6* was decreased and the expression of an NF-κB subunit *RELA* and *CXCL8* was elevated in a subset of samples treated with SW-D-P3, depending on the milk source. In LPS-challenged cells, the expression of *CXCL8* was upregulated and the expression of *IRF5* and *TNFRSF1A* was downregulated in SW-D-P3-treated cells, regardless of animal origin. On the other hand, a number of inflammation-related genes were differentially expressed depending on the animal origin of the samples. Moreover, the higher *IL10* expression observed in cells treated with ovine/caprine SW-D-P3 compared to those treated with SW-D-P3 of bovine, caprine, or ovine origin suggests an anti-inflammatory response, in which alternatively activated macrophages (M2 polarization phenotype) may participate. Overall, these findings suggest that incorporating SW into the food industry, either as a standalone ingredient or supplement, may help to prevent inflammation-related diseases.

## 1. Introduction

Fermented dairy products are widely consumed globally, and their popularity has significantly increased in recent years, with market trends indicating further growth. Consumers are interested in these products due to their nutritional and health benefits [1]. Sweet whey (SW), a by-product of the cheese-manufacturing industry, is produced by enzymatic coagulation usually using chymosin [2,3]. All over the world, cheese plants generate great amounts of SW that can be utilized as an alternative source of bioactive peptides in the nutraceutical and food industries [4]. Within this context, there are several factors influencing the concentration of whey protein in SW including the milk source [5].

Environmental concerns regarding SW stem from its high biological oxygen demand, elevated organic load, and the large quantities generated, all of which contribute to its significant pollutant impact [6]. From a nutritional aspect, SW has nutritional added value, since it contains about 93–94% of water and the following nutrients from the original milk: lactose, soluble proteins, minerals, lactic acid, and fats [7]. On top of that, the protein content of SW has heightened interest in its potential valorization. The increased utilization of whey products is supported by several factors, including the marketability of products labeled as ‘generally recognized as safe’ (GRAS) by food and drug regulatory agencies [8,9].

It is well known that proteins of animal origin are essential for human health because they provide all the necessary amino acids required by the body. In milk, the main proteins are caseins and whey proteins. Within whey proteins, β-lactoglobulin and α-lactalbumin are the most prevalent, while other proteins such as bovine serum albumin, immunoglobulins, lactoferrin, lactoperoxidase, proteose peptones, and various enzymes are present in smaller amounts [10]. Far beyond their nutritional role, whey proteins are a significant source of biologically active components, including bioactive peptides with various health-promoting effects, making them the focus of extensive research [11]. The most extensively studied biological functions of these peptides include cytomodulatory, antithrombotic, and opioid-like effects, mineral-binding capability, antihypertensive and cholesterol-lowering activity, and antimicrobial and antioxidant properties, as well as immunomodulatory effects [12,13,14,15,16,17,18]. It is important to note that although several studies have reported on the immunomodulatory properties of milk whey, most of them use whey protein concentrate (WPC) and whey protein isolate (WPI) derived from bovine milk, rather than fresh SW. Additionally, there is a gap in comparative research on the immunomodulatory properties of fresh SW sourced from lactating ruminants.

Bioactive peptides are released from whey proteins in the gastrointestinal tract through the action of digestive enzymes like pepsin and pancreatic enzymes. These peptides can be absorbed intact into the bloodstream from the intestinal lumen, potentially serving as novel functional food ingredients involved in immunoregulation [9]. Polypeptides can be broken down by brush-border or cellular peptidases, while low-molecular-weight peptides may remain intact and act directly at the tissue level [19]. Some bioactive peptides can be formed during digestion and resist the action of proteolytic enzymes, while others may be degraded, reducing their bioactivity [20]. Moreover, since SW is produced through a fermentation process, this may further enhance the release of bioactive peptides with anti-inflammatory activity [21,22]. Overall, growing evidence supports the idea that whey peptides have a great impact on human health, including their immunomodulatory potential [23].

In the past few years, in vitro digestion methods combined with cell culture models, either 2D or 3D, have gained prominence when studying interactions between dietary components and immune cells, thus offering a more reliable representation of in vivo gastrointestinal conditions, minimizing ethical concerns and facilitating high-throughput studies under well-controlled conditions [24,25]. Additionally, one more valuable model for investigating these interactions is the integration of ex vivo human peripheral blood mononuclear cells with an in vitro digestion method [26]. The innate immune system serves as the body’s first non-specific line of defense and recruits various cell types, including monocytes and macrophages as well as neutrophils and dendritic cells [27]. Inflammation is an important physiological response of the body mediated by immune cells against injury and tissue damage [28]. In the inflammatory process, some conserved immune receptors are involved, such as type I transmembrane receptors including Toll-like receptors (TLRs), which in turn trigger the production and release of various small molecules such as cytokines and chemokines [29].

From a molecular perspective, TLRs are key pattern recognition receptors (PRRs) that play a critical role in initiating the innate immune response by detecting pathogen-associated molecular patterns (PAMPs), such as lipopolysaccharides (LPSs) derived from Gram-negative bacteria [30,31]. Upon recognizing these molecules, TLRs activate intracellular signaling cascades, predominantly through the nuclear factor kappa-light-chain-enhancer of activated B cells (NF-κB) pathway [32]. This activation promotes the nuclear translocation of transcription factors, which induce the expression of immune-related genes, including those encoding cytokines, chemokines, and co-stimulatory molecules essential for pathogen elimination [33,34,35,36]. Among the TLRs, TLR4 is notable for its ability to recognize LPS, while TLR2 has also been implicated in LPS signaling in some contexts [37].

The NF-κB pathway is central to inflammatory responses and can be activated via canonical or non-canonical signaling. The canonical pathway (also known as classical), typically downstream of MyD88-dependent mechanisms triggered by TLRs, relies on the activation of the p50 in conjunction with RelA (p65) subunits of NF-κB, which form a dimer that translocates to the nucleus to activate target genes [38]. The non-canonical pathway, in contrast, relies on distinct signaling components and generally functions in a more specialized context. For example, interferon regulatory factors (IRFs) play significant roles in this pathway, with IRF5 being a key transcription factor promoting the pro-inflammatory M1 macrophage phenotype [39,40]. Strong evidence supports the notion that MyD88-dependent signaling activates IRF5, whereas MyD88-independent mechanisms involve IRF3 activation [30].

Upon NF-κB pathway activation and the translocation of its transcription factors to the nucleus, the next step involves the expression of inflammatory genes, leading to the secretion of cytokines and chemokines. These molecules are critical for immune cell recruitment and activation. TLR4, for instance, leads to the expression of intercellular adhesion molecule-1 (ICAM-1) and key pro-inflammatory cytokines such as tumor necrosis factor-alpha (TNF-α) and interleukin 1-beta (IL-1β) [41,42]. These molecules regulate immune cell recruitment and activation through the production of chemokines like C-X-C motif chemokine ligand 8 (CXCL8) [5]. CXCL8, also known as IL-8, is an evolving chemokine with diverse functions, primarily involved in inflammation and immune responses. One of its key roles is angiogenesis, which is crucial for wound healing and tissue repair but can also promote tumor growth. Additionally, IL-8 plays a significant role in immune cell regulation, influencing various immune cells such as monocytes, lymphocytes, and endothelial cells, contributing to both acute and chronic inflammation [43].

TNF Receptor Superfamily Member 1A (TNFRSF1A) is one of the major receptors for TNF-α, which is involved in the activation of the NF-κB pathway and may be an important biomarker of the inflammation process [44]. Also, it is well established that cell surface molecules, including ICAM-1, interact with specific counterparts, facilitating adhesion to the endothelium and playing a crucial role in pathogenesis and atherosclerosis [45]. Additionally, interleukin 6 (IL-6), a pro-inflammatory cytokine secreted by monocytes and macrophages, serves as a biomarker of inflammation. IL-6 contributes to inflammation progression and endothelial dysfunction, processes that can lead to atherosclerosis [46,47]. Meanwhile, anti-inflammatory cytokines such as interleukin 10 (IL-10) and multifunctional cytokines such as transforming growth factor-beta (TGF-β) can modulate the inflammatory response through suppression or inhibition, balancing the effects of pro-inflammatory mediators. In more detail, IL-10 suppresses the expression of cytokines, including IL-1β and TNF-α, while TGF-β has context-dependent roles in immune regulation and monocyte/macrophage function [48,49].

Overall, macrophages exhibit remarkable plasticity, displaying distinct functional phenotypes in response to environmental signals. The classical dichotomy categorizes macrophages into pro-inflammatory M1 and anti-inflammatory M2 phenotypes [50,51]; however, macrophages exist in a range of activation states, each characterized by unique gene expression profiles and functional roles [40,52]. These properties make the THP-1 model ideal for investigating macrophage responses to inflammatory stimuli and evaluating the immunomodulatory potential of nutrients.

Previous studies have focused on the immunomodulatory effects of dairy by-products, indicating that whey proteins exert anti-inflammatory effects through multiple endogenous pathways. Evidence suggests that whey can modulate cytokine production and NF-κB signaling, thereby contributing to its immunomodulatory effects, although research on its mechanisms remains limited [53]. This study aims to evaluate the effects of SW from various animal sources on inflammatory markers in THP-1 macrophages.

## 2. Results and Discussion

To evaluate the effect of digested SW on the inflammation cascade, THP-1 cells differentiated to macrophages by PMA were treated with fractions of SW-Ds with or without LPS stimulation. Macrophages were once thought to act in a solely pro-inflammatory manner; however, recent studies have revealed that they also play a role in anti-inflammatory processes, making them effective regulators of immune function [54]. Thus, the gene expression of a wide range of transcription factors (*NFKB1*, *RELA*, *IRF5*), receptors (*TLR2*, *TLR4* and *TNFRSF1A*), pro- and anti-inflammatory cytokines (*IL1B*, *IL6*, *IL10* etc.), adhesion molecules (*ICAM1*), and chemokines (*CXCL8*) was evaluated.

### 2.1. Effect of SW-D-P3 on mRNA Expression of Inflammation-Related Genes in Non-Challenged PMA-Induced THP-1-Derived Macrophages

**Figure 1:**
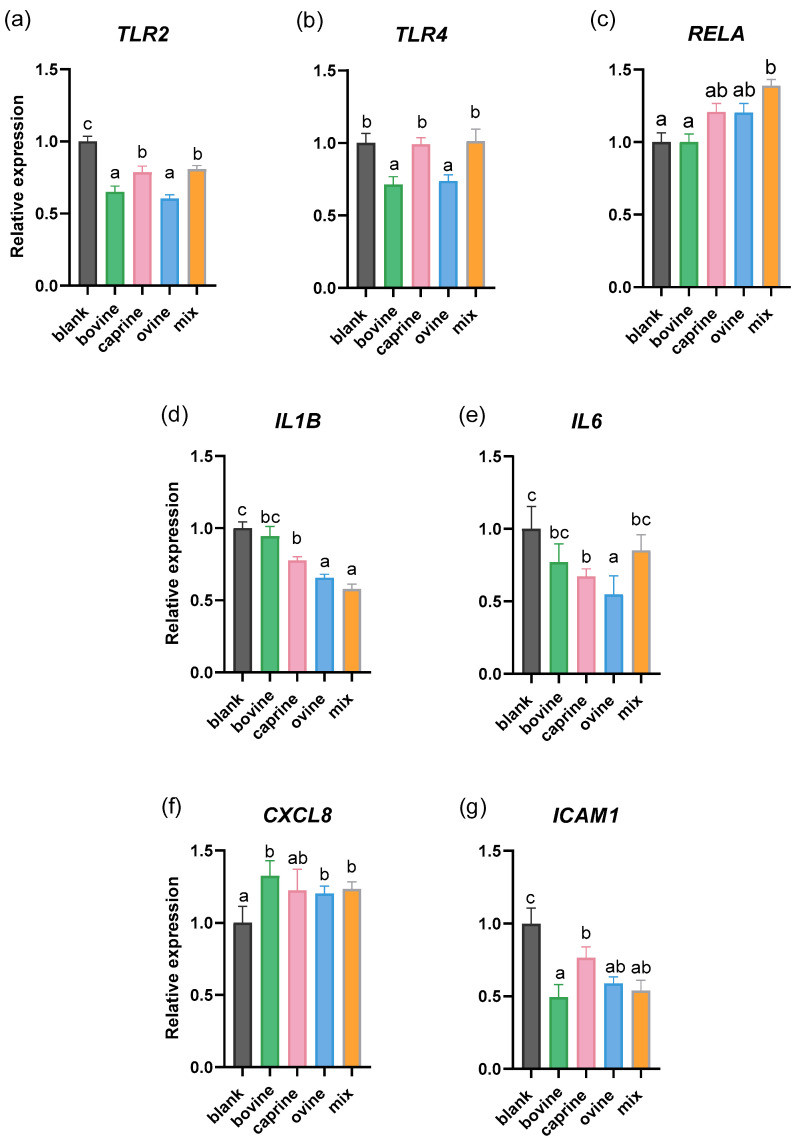
Effect of SW on the mRNA expression of non-challenged PMA-induced THP-1-derived macrophages. THP-1 cells were pre-treated with PMA for 48 h (100 ng/mL), allowed to rest for 24 h, and then treated with D-P3 (0.038% *w*/*v*) or BL-D-P3 for 24 h. (**a**) *TLR2*, (**b**) *TLR4*, (**c**) *RELA*, (**d**) *IL1B*, (**e**) *IL6*, (**f**) *CXCL8,* and (**g**) *ICAM1* gene expression levels measured by qPCR. Data are represented as means ± SEM of three technical replicates, as cell treatments were performed in triplicate. Columns with different letters within the same panel are significantly different (*p* < 0.05).

Previous studies indicated that milk bioactive peptides exhibit anti-inflammatory effects by suppressing NF-κB pathway activation through a peroxisome proliferator-activated receptor gamma (PPARγ)-dependent mechanism. They also modulate the expression of chemokine receptors and TLRs in monocytes/macrophages [55]. In our study, significantly different effects of SW-D-P3 on TLR gene expression upon PMA stimulation were observed. Specifically, *TLR2* expression was found to be lower in SW-D-P3-treated cells when compared to BL-D-P3 (digestion with water instead of sweet whey and corresponding to the fraction of digestate with peptides with a molecular weight below 3 kDa), regardless of the milk’s animal origin. This decrease, however, was even greater in the bovine and ovine samples when compared to the caprine and mixed ones (*p* < 0.05; Figure 1a). *TLR4* expression in turn was decreased only in cells treated with bovine and ovine SW when compared to BL-D-P3 (*p* < 0.05; Figure 1b). The expression of *RELA* (NF-kappa-B p65 subunit) was found to be higher only in mixed SW-D-P3-treated PMA cells when compared either to BL-D-P3 or bovine SW-D-P3 (*p* < 0.05; Figure 1c). Furthermore, ovine and mixed samples downregulated *IL1B* gene expression compared to BL-D-P3, though this effect occurred to a lesser extent for caprine, with no significant difference for the bovine counterpart (*p* < 0.05; Figure 1d). Regarding *IL6*, another key pro-inflammatory biomarker, its expression was observed to be lower in caprine and ovine SW-D-P3-treated cells compared to BL-D-P3, with a more pronounced reduction in macrophages treated with SW from ovine milk origin (*p* < 0.05; Figure 1e). Also, the transcription levels of *CXCL8* were significantly increased (*p* < 0.05; Figure 1f) by bovine, ovine, and mixed SW compared to BL-D-P3, whereas caprine SW did not show this effect in PMA-induced cells. In the context of adhesion molecule expression, the results demonstrate that macrophages treated with SW have decreased ICAM1 mRNA expression (*p* < 0.05; Figure 1g), particularly for SW of bovine origin. No statistical differences were observed between treated cells regarding *NFKB1*, *TGFB1*, *IRF5*, *TNF,* and *TNFRSF1A* expression (*p* > 0.05; Supplementary Materials, Figure S1).

TLRs, including TLR2 and TLR4, can activate the NF-κB signaling pathway, leading to the upregulation of adhesion molecules and pro-inflammatory cytokines [56]. Therefore, we aim to investigate whether SW-D-P3 and any bioactive peptides, released or generated during digestion, can modulate key molecules such as *ICAM1*, *IL1B* and *IL6*. Both bovine and ovine SW-D-P3 significantly inhibited the expression of *TLR2*, *TLR4,* and *ICAM1* (Figure 1a,b,g, respectively) in the M0 phenotype. However, only ovine SW-D-P3 reduced the transcription levels of *IL1B* and *IL6* (Figure 1d,e, respectively). It is interesting to note that Kiewiet et al. [57] observed that the effects of cow’s milk proteins on the immune system vary depending on the type of protein and the level of hydrolysis, with TLR signaling proposed as a potential mechanism underlying these differences. This hypothesis could explain the effects of milk origin observed in the present study, where the stronger immunomodulatory activity of ovine D-P3 may be attributed to variations in whey protein sequences between species and differences in the degree of hydrolysis, which could result from both intrinsic sequence differences and the effects of fermentation or digestion.

In our study, the observed increase in *CXCL8* gene expression supports its established role in inflammation and immune regulation. Consistent with our findings, Nguyen et al. observed that WPC powders, under different thermal processing conditions and whey sources (sweet or acid whey), induced higher IL-8 secretion in porcine intestinal epithelial cells compared to the control group [58]. However, the differences are likely due to the protein denaturation methods (pasteurization and spray drying) rather than a result of the in vitro digestion in our study. Taken together, these findings suggest that despite the observed downregulation of *TLR2* and *TLR4*, the upregulation of *RELA* and *CXCL8* may be attributed to TLR-independent mechanisms. These genes can be induced independently of feedback signaling, as NF-κB and activating protein-1 (AP-1) activation is governed by multiple regulatory pathways beyond TLR signaling. While TLRs activate the conserved MyD88-dependent pathway, leading to NF-κB and AP-1 activation, these transcription factors can also be modulated by cytokine-mediated responses and non-canonical NF-κB signaling [34]. This suggests that the inflammatory response observed may be the result of a broader regulatory network rather than being exclusively driven by TLR2/TLR4 activation.

Milk and dairy products, including whey and casein, have been shown to influence adhesion molecules like ICAM-1. A previous study suggested that the consumption of whey protein can lead to a significant decrease in the soluble form of ICAM-1, potentially improving vascular reactivity [59]. This effect aligns with our findings, which highlighted a downregulation of *ICAM1* from all SW tested regardless of animal origin. This is of great interest as another previous study has demonstrated that *ICAM1* expression is an early event in the development of atherosclerotic lesions, as it is closely linked to angiogenesis and neovascularization [60]. Therefore, the results of the present study could position SW as a potential agent for the prevention of atherosclerotic events.

Our findings in conjunction with previous reports suggest that the degree of hydrolysis plays a role in the effects of SW on cytokine expression in THP-1 macrophages. While our study observed modulation of the gene expression of several cytokines, *TNF* was not one of them, constituting an exception. This is in contrast with numerous previous studies [53,61,62], which reported that whey-derived peptides can either inhibit or promote *TNF* expression. Importantly, those studies predominantly utilized pre-hydrolyzed WPC and/or WPI as starting materials, differing from the degree of hydrolysis applied in our approach. These findings underscore how varying levels of hydrolysis between studies can influence the immunomodulatory activity of SW on inflammatory gene expression in differentiated THP-1 macrophages.

### 2.2. Effect of SW-D-P3 on mRNA Expression of Inflammation-Related Genes in LPS-Challenged PMA-Induced THP-1-Derived Macrophages

**Figure 2:**
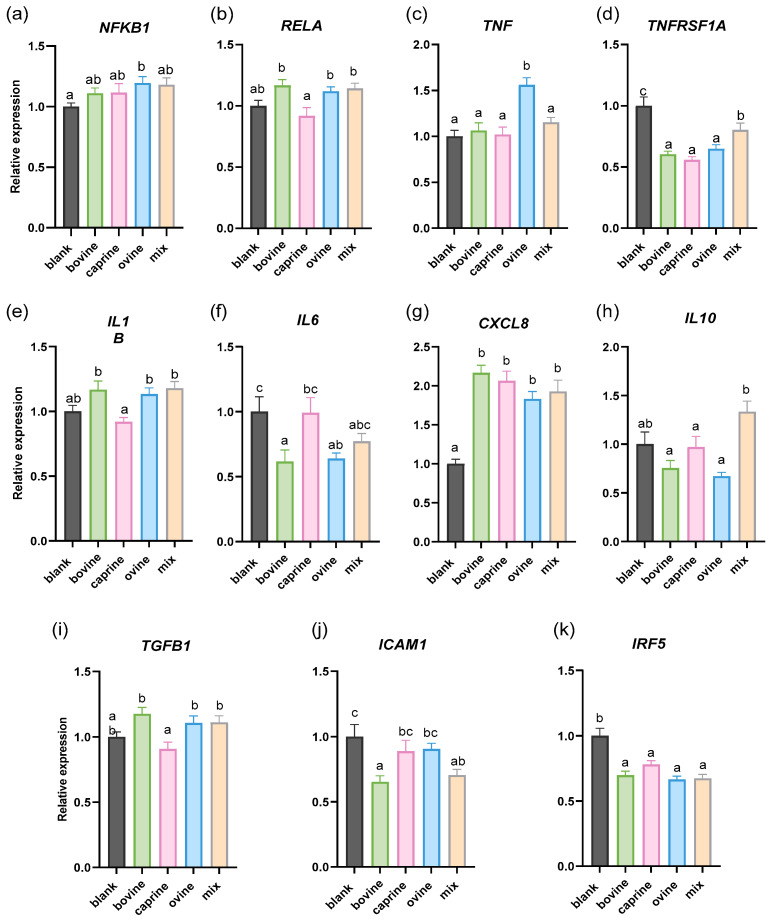
Effect of SW on the mRNA expression in LPS-challenged PMA-induced THP-1-derived macrophages. THP-1 cells were pre-treated with PMA for 48 h (100 ng/mL), allowed to rest for 24 h, and then treated with LPS (100 ng/mL) in the presence of SW-D-P3 (0.038% *w*/*v*) or BL-D-P3 for 24 h. (**a**) *NFKB1*, (**b**) *RELA*, (**c**) *TNF*, (**d**) *TNFRSF1A*, (**e**) *IL1B*, (f) *IL6*, (**g**) *CXCL8*, (**h**) *IL10*, (**i**) *TGFB1*, (**j**) *ICAM1,* and (**k**) *IRF5* gene expression levels were measured by qPCR. Data are represented as means ± SEM of three technical replicates, as cell treatments were performed in triplicate. Columns with different letters within the same panel are significantly different (*p* < 0.05).

In order to gain a deeper insight into the effect of SW on human monocyte inflammation, we next examined their effect on gene expression in LPS-stimulated THP-1 macrophages. LPS stimulation at 100 ng/mL was used to activate THP-1 differentiated macrophages, promoting M1 polarization and triggering low-grade inflammation. Firstly, Figure 2a shows an increase in *NFKB1* expression by ovine SW-D-P3 treatment compared to BL-D-P3. No significant difference in the *RELA*, *IL1B,* and *TGFB1* mRNA expression (Figure 2b,e,i, respectively) was observed between SW-D-P3 and BL-D-P3. Interestingly, caprine SW-D-P3 displayed lower transcriptional activity of these genes (*RELA*, *IL1B*, and *TGFB1*) when compared to bovine, ovine, and mixed SW-D-P3. Next, as shown in Figure 2c, LPS stimulation of THP-1-derived macrophages resulted in an increase in the mRNA levels of *TNF* only for bovine SW-D-P3, while a decrease in *ICAM1* was observed in both bovine and mixed samples (Figure 2j). Furthermore, as shown in Figure 2g, cells treated with SW-D-P3 showed significantly higher levels of *CXCL8* compared to those treated with BL-D-P3, regardless of the animal origin of the samples. In contrast, the opposite pattern was observed for *TNFRSF1A* and *IRF5* (Figure 2d,k, respectively). Regarding *IL10* expression, it was found to be upregulated in cells treated with mixed SW-D-P3 when compared to the other SW-D-P3s but not with the blank digest control (Figure 2h). Finally, a significant downregulation of *IL6* was observed in cells treated with bovine and ovine SW-D-P3 compared to those treated with BL-D-P3 (*p* < 0.05; Figure 2f). No statistical differences were observed between treated cells regarding *TLR2* and *TLR4* expression (*p* > 0.05; Supplementary Materials, Figure S2). The absence of regulation of TLRs in LPS-challenged conditions may seem contradictory to the observation made in non-challenged conditions. However, LPS exposure elicits a strong pro-inflammatory response, sustaining or even upregulating TLR expression to support immune activation, thus counteracting any possible regulatory effects from SW-D-P3. Therefore, under non-challenged conditions, the absence of this pro-inflammatory signaling may allow SW-D-P3 to promote TLR downregulation more effectively.

In a previous study by Kanwar et al. [63], digested WPC was evaluated in a more complex cell culture model, specifically a transwell co-culture system involving PMA-induced, LPS-challenged THP-1 macrophages and Jurkat human T lymphocytes. Cytokine secretion analysis showed an upregulation in the secretion of the anti-inflammatory cytokine IL-10, which may be beneficial in reducing the effects of chronic gut inflammatory diseases such as inflammatory bowel disease. To further explore the hypothesis that SW has anti-inflammatory properties, *IL10* expression (an M2 macrophage marker) was evaluated only in the LPS-challenged model. While previous studies [64,65] focusing on whey protein hydrolysate have reported increased IL-10 production in LPS-stimulated THP-1 cells, these findings were based on comparisons to different controls (non-treated LPS-stimulated cells) than those used in the present study. Using a more rigorous control (blank digest), our findings do not support an IL-10-mediated anti-inflammatory effect of SW. However, the observed differences between samples of different animal origins are noteworthy. Specifically, mixed-origin samples appear to exert a more favorable effect on *IL10* modulation, suggesting potential variability in SW’s immunomodulatory properties depending on its composition.

Regulation of the NF-κB pathway is associated with the expression of both pro-inflammatory (e.g., *IL6*, *TNF*, *IL1B*) and anti-inflammatory genes (e.g., *IL10*). In a recent study by Han et al., protein hydrolysates derived from oilseed proteins were compared with the two dairy bovine protein fractions, whey and casein, using an LPS-stimulated murine macrophage cell line and the fraction with molecular weight 0–3 kDa [66]. In line with our results, bovine whey was unable to suppress the gene expression of *TLR4*, *TNF*, *IL1B*, *IL10*, *NFKB1,* and *RELA*. On the other hand, *IL6* was not affected by treatment with whey digest fractions in contrast with our finding that treatment with bovine SW-D-P3 resulted in the downregulation of *IL6*. TNFRSF1A is the key cell surface receptor for the cytokine TNF-α. When TNF-α binds to its receptor, it induces activation of the transcription factor NF-κB. In our study, cells treated with SW-D-P3 showed decreased *TNFRSF1A* gene expression compared to those treated with BL-D-P3, regardless of the animal origin of samples. In a previous study, Gjevestad et al. [67] investigated whether the intake of protein-enriched milk for 12 weeks would influence markers of inflammation among elderly people. In line with our results, the mRNA level of *TNFRSF1A* was significantly reduced in isolated ex vivo PBMCs, highlighting the correlation between the intake of dairy products and activation of the NF-κB pathway.

Li et al. [68] evaluated NF-κB inhibition at the protein level using RAW 264.7 and THP-1 cells cultured with LPS and treated with digested donkey and bovine whey proteins. They found that only donkey whey proteins suppressed LPS-induced NF-κB DNA-binding activity. This aligns with our findings, where no difference was observed in *NFKB1* or *RELA* expression in LPS-stimulated THP-1 macrophages treated with bovine SW. However, differences in digestion, cell culturing, and controls used should be noted.

In the present study, the expression of *TLR2* and *TLR4* showed no significant differences across the various milk origins of SW at a concentration of 0.38 mg/mL in LPS-activated THP-1 cells. These results align with those reported in a previous study [69], in which the effect of bovine whey fraction was evaluated on *TLR2* and *TLR4* gene expression in human enterocyte Caco-2/TC7 cells. Specifically, cells treated with a comparable concentration of 0.5 mg/mL of whey did not show any significant changes in *TLR2* and *TLR4* mRNA expression. Conversely, the expression of these receptors was significantly affected at the highest concentration of 10 mg/mL. Furthermore, another study [70] reported that WPI (0.1 mg/mL) pre-treatment did not significantly inhibit the LPS-induced expression of inflammatory genes, including *TLR4*, *NFKB1,* and *TNF*, in HT29-MTX goblet cells. However, the correlation is indirect, as factors such as differences in the starting material, cell type, LPS concentration, absence of digestion, and comparison with LPS treatment rather than the blank digest should be considered.

In a previous study, Olsen et al. observed that in vitro-digested bovine WPI exhibited a dose-dependent effect, with treatment doses of 10 µg/mL, 100 µg/mL, and 1000 µg/mL leading to a progressively greater reduction in IL-1β production at higher concentrations. [36]. In our study, cells treated with sweet whey did not show any significant changes in *IL1B* mRNA expression. However, we consider it unlikely that a direct correlation can be drawn between the results, as WPI is a non-fat whey protein isolate, extracted using membrane filtration, and it is a commercially available product. This makes it a more purified form compared to the SW samples used in our study, which were collected directly from production in bulk. Moreover, the observed reduction refers to an LPS-stimulated control and not a blank digest control that was used in our study. In the same context, LPS-stimulated porcine colonic tissue was co-incubated with a peptide-enriched fraction of 1 kDa permeate of casein hydrolysate for 3 h, and the expression of a panel of inflammatory cytokines was measured using qPCR. No significant differences were observed in the expression of *IL1B*, *TNF,* and *TGFB1* between the permeate fraction and the control group [71]. It is important to note that the main distinctions in this study lie in the presence of macrophages beneath the mucosal layer of the intestine, as well as the use of casein rather than whey.

Previous studies explored the potential anti-inflammatory peptides derived from Bing-langjiang buffalo whey protein hydrolysate. Three novel peptides (DQPFFHYN, YSPFSSFPR, and GPGAPADPGRPTG) were screened out using LPS-stimulated RAW264.7 macrophages. The findings revealed that peptides DN8 and YR9 effectively suppressed the secretion of pro-inflammatory cytokines TNF-α and IL-6, as well as the expression of *TNF* and *IL6* in inflammatory macrophages [72]. Additionally, the peptide GG13 inhibited *TNF* expression and under identical culture conditions [73]. In our study, we focused on the 3 kDa fraction from SW, which contains naturally derived bioactive compounds, while synthetic peptides offer greater purity, specificity, and bioavailability. Given the complex composition of the 3 kDa fraction, its effects may be less predictable than those of synthetic peptides, but it provides valuable insights into the broader bioactivity of whey-derived components.

A key observation in the current study was that *CXCL8* expression was increased in response to digested SW, regardless of the milk source, both with and without LPS stimulation. To our knowledge, this effect of digested SW on *CXCL8* has not been previously observed in THP-1 cells. Results from previous studies have reported a decrease in IL-8 secretion following treatment with digested whey proteins in human respiratory cell lines followed by 2.5 μg/mL LPS challenge [74] and following treatment with in vitro-digested human milk in 0.1 μg/mL LPS-challenged THP-1 cells [75]. However, the differences observed regarding the regulation of *CXCL8* expression in the present study may stem from a variety of factors, with the intensity of the challenge being one of the most significant. This is particularly highlighted by the differences in the concentrations of LPS used between the studies. Variations in LPS concentration can lead to differing levels of receptor activation, signaling pathways, and ultimately, the expression of *CXCL8*, thereby influencing results.

Human and animal intervention trials involving diets that include whey products are the most effective way to assess their potential immunomodulatory benefits. However, only a limited number of such studies have examined the immunomodulatory effects of whey proteins or peptides. In a previous study, Menahem et al. [76] observed no changes in the gene expression levels of *IL1B* and *TNF* in the spleens of young rats fed either a soy-based or whey-based diet after being injected with LPS to induce subclinical inflammation. Another study utilized a randomized three-way crossover design involving twenty overweight and obese postmenopausal women. Participants consumed a breakfast meal with one of two supplements, WPI or casein. This study found no significant acute effect on plasma inflammatory markers, including IL-6 and TNF-α [77]. Consistent with previous research, the effects of a novel whey-derived peptide on vascular endothelial function in healthy young men and women showed no significant changes in inflammation markers [78]. In contrast, another study has shown that feeding with fermented whey may help prevent inflammation following the induction of colitis. Specifically, pre-consumption of whey fermented with the probiotic *Lactobacillus rhamnosus* prior to colitis induction significantly reduced pro-inflammatory markers (IL-4 and TNF-α) and increased levels of the anti-inflammatory marker TGF-β in the intestine [79]. Consequently, although, in our study, some modulation of inflammation-related genes by SW-D was reported, the in vivo effects were less clearly defined. This underscores the need for further research to gain a deeper understanding of the complex interactions between whey products and immune responses, as current research has not consistently demonstrated pronounced differences or effects.

At this point, it is important to highlight that both the exposure time and the concentration of LPS are key factors in understanding the mechanisms that drive inflammation, particularly concerning cytokine release regulation [80]. Specifically, the incubation time with LPS can significantly affect the magnitude and type of inflammatory response elicited. Moreover, the concentration of LPS is crucial in determining the intensity of this response; lower concentrations may trigger a mild inflammatory response, often referred to as low-grade inflammation, while higher concentrations can lead to robust and potentially dysregulated cytokine release.

The potential clinical benefits of caprine and ovine SW in reducing the expression of pro-inflammatory cytokines, particularly *IL1B* and *IL6*, suggest a promising opportunity for managing inflammation-related conditions. Further research is necessary to clarify the mechanisms by which in vitro-digested SW mediates these anti-inflammatory effects.

## 3. Materials and Methods

### 3.1. Chemicals and Reagents

The following chemicals, enzymes, and reagents used were of high purity and analytical reagent grade. Pepsin from porcine gastric mucosa (≥2.500 units/mg protein), porcine pancreatin (4 × USP, United States Pharmacopeia), porcine bile extract, phorbol 12-myristate 13-acetate (PMA) and lipopolysaccharides from *Escherichia coli* O111:B4 (LPSs) were obtained from Sigma-Aldrich (Saint Louis, MO, USA). The Amicon Ultra-4 Centrifugal Filter Devices (3 kDa and 10 kDa) and Millex-GP 33 mm PES 0.22 um were purchased from Millipore (Burlington, MA, USA). The 96-well cell culture transparent flat-bottom plates were purchased from Kisker Biotech (Steinfurt, Germany), while cell culture flasks and plates were purchased from SPL Life Sciences (Pocheon, Republic of Korea). Fetal bovine serum was obtained from Gibco ThermoFisher Scientific (Waltham, MA, USA). RPMI 1640, L-glutamine, penicillin–streptomycin, sodium pyruvate, and non-essential amino acids were obtained from Biosera (Cholet, France), while 3- (4,5-dimethylthiazol2-yl)-2,5-diphenyltetrazolium bromide (MTT) reagent was purchased from Cayman (Michigan, MI, USA). NucleoZOL was purchased from Macherey-Nagel (Düren, Germany). DNase I (RNase- Free) was from New Englands Biolabs (Ipswich, MA, USA), while PrimeScript RT Reagent Kit (Perfect Real Time) and phosphate-buffered saline (PBS) were from Takara Bio (Shiga, Japan). The FastGene IC Green 2 × IC Green qPCR Universal Mix was obtained from Nippon Genetics (Tokyo, Japan).

### 3.2. Collection and Preparation of Sweet Whey

Following a thorough search for available SW samples across Greece, 48 different samples were obtained from several small-scale cheese plants. Cheeses were produced using milk from bovine, ovine, caprine, and an ovine/caprine mixture. For the mixed samples, denoted as “SW mix” hereafter, the ovine/caprine milk ratio varied between 80/20 and 70/30. An equal number of SW samples (*n* = 12) from each milk origin (bovine, ovine, caprine, and an ovine/caprine mixture) were used in this study. The liquid SW samples were freeze-dried to remove water and other solvents, and protein content was subsequently assessed by the Kjeldahl method in duplicates [81]. The protein content of SW ranged from 0.3 to 2% *w*/*v*, and pH ranged from 4.5 to 6.5.

### 3.3. Simulated Gastrointestinal Digestion and Digestates’ Fractionation

All SW samples were concentrated ten-fold by freeze-drying, followed by rehydration. The freeze-drying process was conducted under temperature conditions ranging from −20 °C to 15 °C, with the temperature increasing by 5 °C every 4 h. The maximum temperature difference between the shelf and the sample was 10°C, and the vacuum pressure was maintained at 1 mbar throughout the procedure. At that point, all SW samples were resuspended in water to achieve the same protein concentration of 3% (*w*/*v*), since digestion was performed on the basis of equal protein amounts.

Simulated gastrointestinal digestion followed the detailed methodology outlined by Dalaka et al. [82]. The digestion procedure was based on the improved method INFOGEST 2.0 [83,84] with slight modifications. This protocol is designed to mimic the conditions of the oral, gastric, and intestinal phases. Amylase and pancreatic lipase were not employed, since our samples do not contain significant amounts of starch and fat, respectively. Electrolyte stock solutions for digestive fluids, including simulated salivary fluid, simulated gastric fluid, and simulated intestinal fluid, were prepared, and their pH levels were adjusted using 5 M HCl and 6 M NaOH. Pepsin, pancreatin, and bile salt solutions were freshly prepared immediately prior to use.

Upon completion of the intestinal digestion phase, the SW digests (SW-Ds) were heated to 85 °C for 10 min and then directly placed on ice. The samples were then centrifuged at 1200× *g* for 5 min, and the supernatants were filtered through 0.22 μm sterile PVDF syringe filters. To obtain fractions with molecular weights between 0 and 3 kDa (SW-D-P3), membrane filters with a molecular weight cut-off of 3 kDa were used. All samples were subsequently stored at −20 °C until further analysis. Digestion was performed in duplicate. Additionally, four replicates of blank digests were prepared in parallel using water instead of SW, following the same in vitro digestion protocol. The resulting blank fraction is hereby referred to as BL-D-P3 in the text and as blank in the corresponding diagrams. SW-Ds had 0.38% *w*/*v* protein concentration as described previously [85].

### 3.4. THP-1 Cell Culture, Cell Viability, Differentiation and Activation

THP-1 cells, derived from a human acute monocytic leukemia cell line, were cultured in RPMI 1640 medium supplemented with 10% (*v*/*v*) fetal bovine serum, 10 U/mL L-glutamine, 1 mM sodium pyruvate, 100 U/mL penicillin, 100 μg/mL streptomycin, and 100 μM non-essential amino acids and were maintained in a humidified incubator at 37 °C with 5% CO2. Similarly to previous research, we adhered to the established protocol to induce differentiation into macrophage-like cells by incubating monocytes with PMA [50,86] and afterwards, macrophages were co-incubated in the presence of LPS and D-P3 [87]. In detail, monocytes were seeded in 6-well plates at a density of 8 × 10^5^ cells/mL, using 2.5 mL per well, and treated with 100 ng/mL PMA for 48 h. After this period, the medium was discarded, the attached cells were washed, followed by an incubation with PMA-free supplemented RPMI-1640 medium for an additional 24 h (resting phase). Subsequently, macrophages were exposed to SW-D-P3 or BL-D-P3 (10%) for 24 h with or without 100 ng/mL LPS. Cell treatments were performed in triplicate.

### 3.5. Quantification of Gene Expression in THP-1 Cells

Following treatment, total RNA was extracted from the attached cells using the NucleoZOL reagent according to the manufacturer’s instructions. Genomic DNA was eliminated with DNase I (20,000 Units/mg) according to the manufacturer’s protocol, and pure RNA was recovered through ethanol precipitation [88]. RNA quantity and purity were assessed using a spectrophotometer (Q5000, Quawell Technology Inc., San Jose, CA, USA). Reverse transcription was then carried out using the PrimeScript RT reagent kit (Takara) following the manufacturer’s protocol. A thermal cycler (SaCycle96, Sacace Biotechnologies, Como, Italy) was used for the qPCR reactions using the FastGene 2 × IC Green qPCR Universal Mix. Each reaction was conducted in duplicate. Primers for target genes and housekeeping genes (*B2M*, *RPL37A*, *RPS18* and *HPRT1*) were designed (by our group) with an annealing temperature of 60 °C. Relative gene expression, normalized to housekeeping genes, was calculated using the method outlined by Hellemans et al. [89]. The primer details are listed in Table 1.

### 3.6. Statistical Analysis

The statistical analysis was conducted using SPSS version 22.0.0, and graphs were generated using GraphPad Prism 8. All data presented are expressed as means ± SEMs of at least two biological replicates. Data underwent a Kolmogorov–Smirnov test and were transformed to logarithmic or normalized forms [90] until a normal distribution was achieved. Afterwards, data comparisons were made using one-way ANOVA followed by Duncan’s post hoc test. Differences at *p* < 0.05 were considered statistically significant.

## 4. Conclusions

The immunomodulatory potential of in vitro-digested SW triggered the NF-κB canonical pathway in a distinct manner depending on the milk source, while simultaneously showing decreased expression of pro-inflammatory cytokines including *IL1B* and *IL6*. Moreover, mixed SW was able to enhance *IL10* transcription levels in LPS-activated THP-1 macrophages. Additionally, SW reduced *ICAM1* in non-challenged as well as LPS-challenged PMA-induced THP-1-derived macrophages, particularly bovine and mixed samples. These findings underline the potential of SW as a valuable functional supplement and provide a strong basis for future research. However, since the current data only support in vitro mechanisms, further in vivo experimental verification is required to confirm its practical applications. These findings provide a theoretical basis for food industry applications but should not be directly extrapolated to in vivo conditions. To support sustainable cheese production, innovative methods for repurposing this by-product are needed. Fermentation and digestion release immunomodulatory compounds, highlighting the opportunity to upcycle SW into valuable products. Further cellular and possibly clinical studies are deemed necessary to understand the impact of milk sources and the peptide sequences derived from them on the inflammation process.

## Figures and Tables

**Table 1:** Oligonucleotide primer sequences, amplicon size, and reaction efficiency in qPCR.

Gene (Accession Number)	Primer Direction	Sequence (5′-3′)	Amplicon Size	Reaction Efficiency
*TLR2* (NM_001318793.2)	Forward	ATCAGCAGGAACAGAGCACA	173	102
	Reverse	ACTCAGGAGCAGCAAGCAC		
*TLR4* (NM_003266.4)	Forward	GATTTATCCAGGTGTGAAATCCAG	174	105
	Reverse	TAGAGATGCTAGATTTGTCTCCAC		
*NFKB1* (NM_001382627.1)	Forward	GATCTGCCAACTACTCCCA	137	92
	Reverse	CCCAGAGACCTCATAGTTGTC		
*RELA* (NM_001145138)	Forward	GGACTACGACCTGAATGCTG	228	105
	Reverse	ACCTCAATGTCCTCTTTCTGC		
*TNF* (NM_000594.4)	Forward	TTCCTCAGCCTCTTCTCCT	196	100
	Reverse	GAGGGTTTGCTACAACATGG		
*TNFRSF1A* (NM_001065.4)	Forward	GTTCCACCTTCACCTCCAG	199	99
	Reverse	GGGTCATCAGTGTCTAGGC		
*IL1B* (NM_000576.3)	Forward	CAGATGAAGTGCTCCTTCCAG	244	99
	Reverse	CCTCGTTATCCCATGTGTCG		
*IL6* (NM_000600.5)	Forward	GGATTCAATGAGGAGACTTGC	205	95
	Reverse	CATTTGTGGTTGGGTCAGG		
*CXCL8* (NM_000584.4)	Forward	GCTAAAGAACTTAGATGTCAGTGC	191	97
	Reverse	AACTTCTCCACAACCCTCTG		
*IL10* (NM_000572.3)	Forward	CATGCTTCGAGATCTCCGAG	122	103
	Reverse	AACCCAGGTAACCCTTAAAGTC		
*TGFB1* (NM_000660.7)	Forward	TGAACCCGTGTTGCTCTC	287	94
	Reverse	TAGTGAACCCGTTGATGTCC		
*ICAM1* (NM_000201.3)	Forward	CAGACCTTTGTCCTGCCA TCGTTGCCATAGGTGACTG	176	95
	Reverse			
*IRF5* (NM_032643.5)	Forward	GGAAATACACCGAAGGCGT ATCCTCTGCAGCTCTTCCT	244	108
	Reverse			
*B2M* (NM_004048)	Forward	GCTATCCAGCGTACTCCA CTTAACTATCTTGGGCTGTGAC	285	103
	Reverse			
*RPL37A* (NM_000998)	Forward	AGTACACTTGCTCTTTCTGTGG GGAAGTGGTATTGTACGTCCAG	119	106
	Reverse			
*RPS18* (NM_022551)	Forward	CTGAGGATGAGGTGGAACG	240	98
	Reverse	CAGTGGTCTTGGTGTGCT		
*HPRT1* (NM_000194)	Forward	CTTTGCTTTCCTTGGTCAGG	111	99
	Reverse	CAAATCCAACAAAGTCTGGCT		

## Data Availability

Data are contained within the article.

## Supplementary Materials

The following supporting information can be downloaded at: https://www.mdpi.com/article/10.3390/molecules30061261/s1, Figure S1. Effect of SW on the mRNA expression of non-challenged PMA-induced THP-1-derived macrophages.THP-1 cells were pretreated with PMA for 48 h (100 ng/mL), allowed to rest for 24 h, and then were treated with LPS (100 ng/mL) in the presence of SW-D-P3 (0.038% w/v) or BL-D-P3 for 24 h. (a) NFKB1, (b) TGFB1, (c) IRF5, (d) TNF and (e) TNFRSF1A gene expression levels were measured by qPCR. Data are represented as means ± SEM of three technical replicates, as cell treatments were performed in triplicate. Figure S2. Effect of SW on the mRNA expression in LPS-challenged PMA-induced THP-1-derived macrophages. THP-1 cells were pre-treated with PMA for 48 h (100 ng/mL), allowed to rest for 24 h, and then treated with LPS (100 ng/mL) in the presence of SW-D-P3 (0.038% w/v) or BL-D-P3 for 24 h. (a) TLR2 and (b) TLR4 gene expression levels were measured by qPCR. Data are represented as means ± SEM of three technical replicates, as cell treatments were performed in triplicate.
