# Supplementary material for: Immunomodulatory Properties of Sweet Whey-Derived Peptides in THP-1 Macrophages
Source: Molecules. 2025 Mar 11;30(6):1261. doi: 10.3390/molecules30061261 (PMC11944360; doi:10.3390/molecules30061261)
Supplement: Supplementary file 1 [file molecules-30-01261-s001.zip › Supplementary figures.pdf]

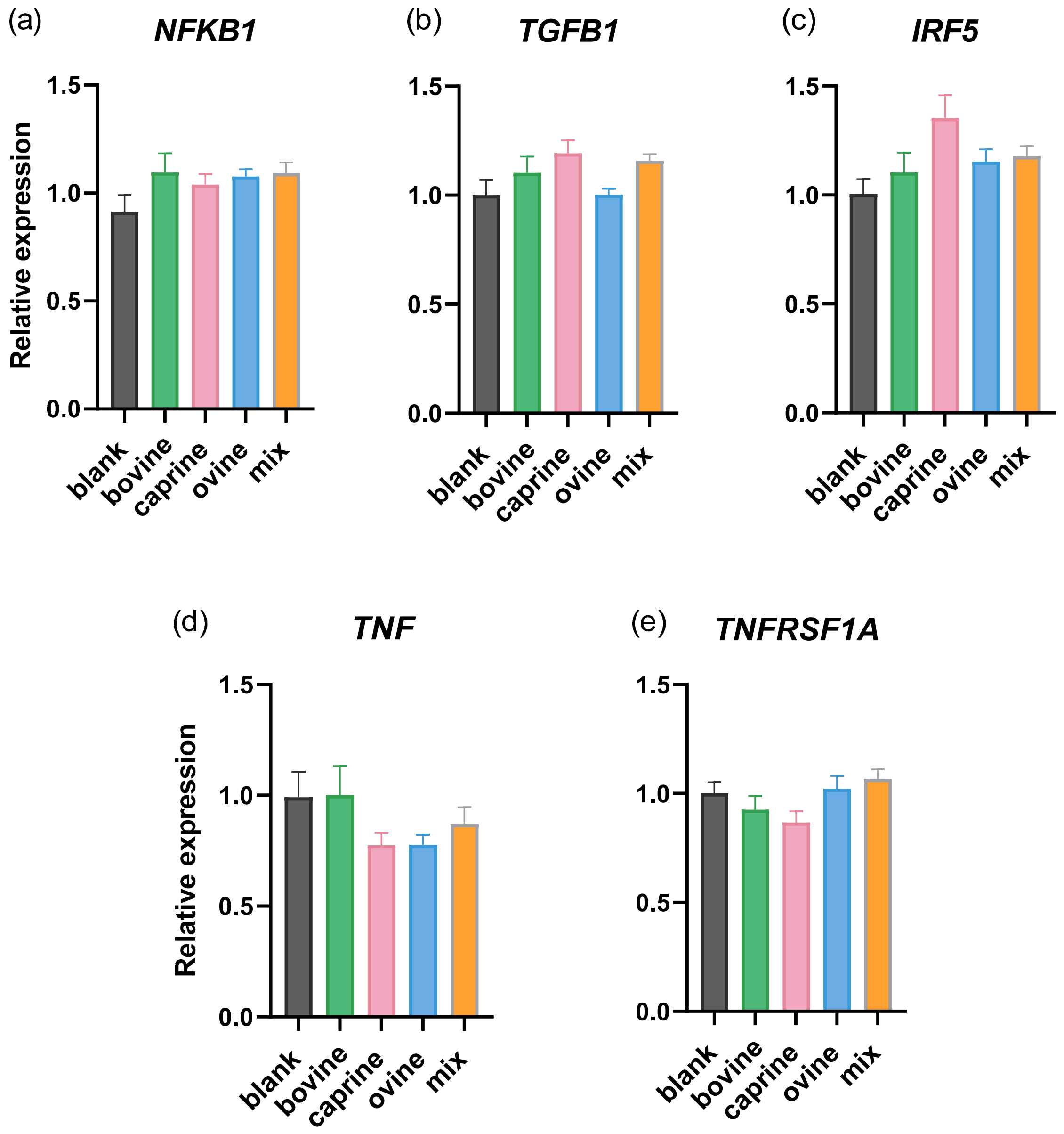

Figure S1. Effect of SW on the mRNA expression of non-challenged PMA-induced THP-1-derived macrophages.THP-1 cells were pretreated with PMA for 48 h (100 ng/mL), allowed to rest for 24 h, and then were treated with LPS (100 ng/mL) in the presence of SW-D-P3 (0.038% w/v) or BL-D-P3 for 24 h. (a) *NFKB1*, (b) *TGFB1*, (c) *IRF5*, (d) *TNF* and (e) *TNFRSF1A* gene expression levels were measured by qPCR. Data are represented as means ± SEM of three technical replicates, as cell treatments were performed in triplicate.

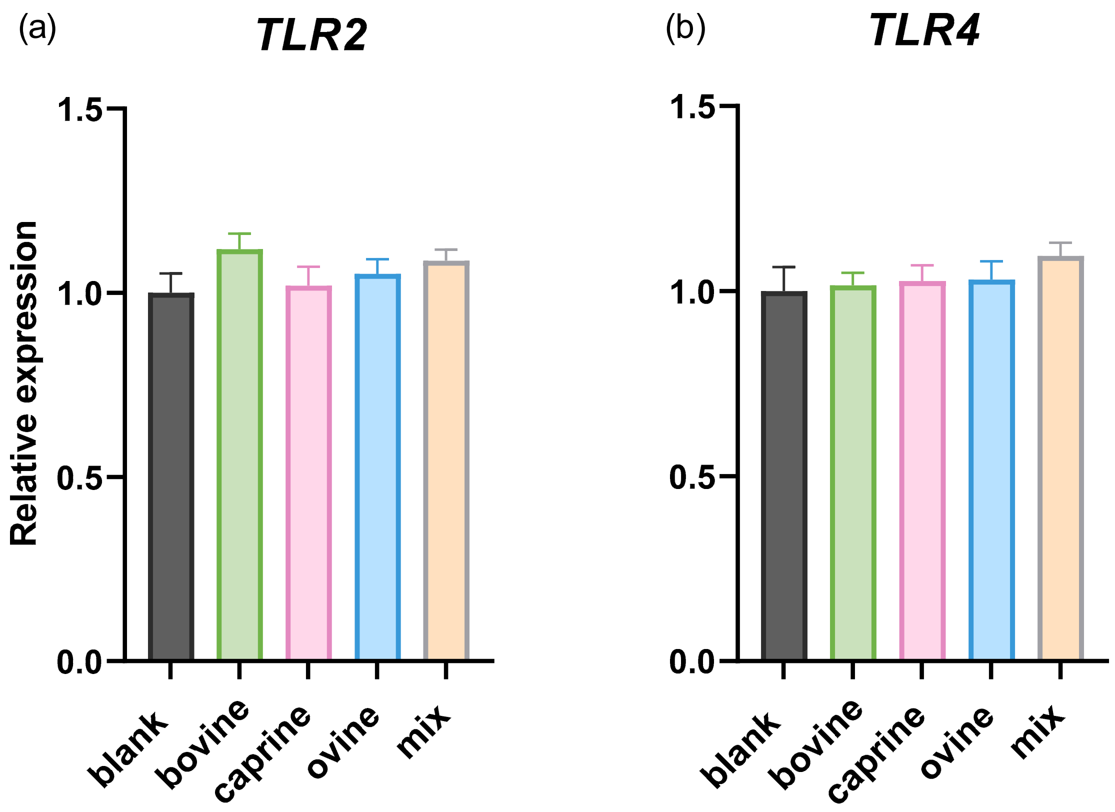

Figure S2. Effect of SW on the mRNA expression in LPS-challenged PMA-induced THP-1-derived macrophages. THP-1 cells were pre-treated with PMA for 48 h (100 ng/mL), allowed to rest for 24 h, and then treated with LPS (100 ng/mL) in the presence of SW-D-P3 (0.038% *w*/*v*) or BL-D-P3 for 24 h. (**a**) *TLR2* and (**b**) TLR4 gene expression levels were measured by qPCR. Data are represented as means ± SEM of three technical replicates, as cell treatments were performed in triplicate.
