# Supplementary figures and images for: Immunomodulatory Properties of Sweet Whey-Derived Peptides in THP-1 Macrophages
Source: Molecules. 2025 Mar 11;30(6):1261. doi: 10.3390/molecules30061261 (PMC11944360; doi:10.3390/molecules30061261)

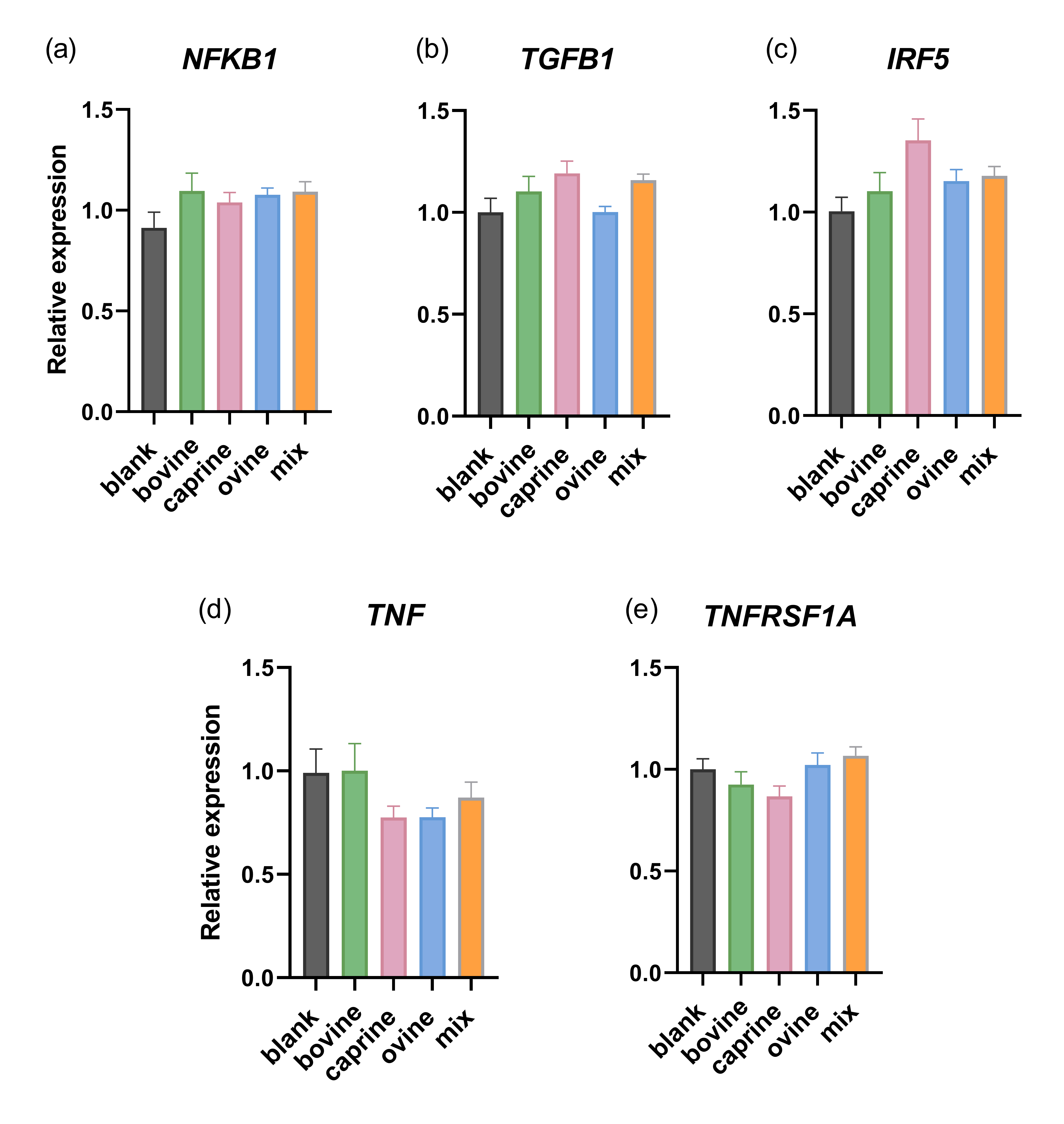

Supplement: Supplementary file 1 [file molecules-30-01261-s001.zip › Figure S1.tif]

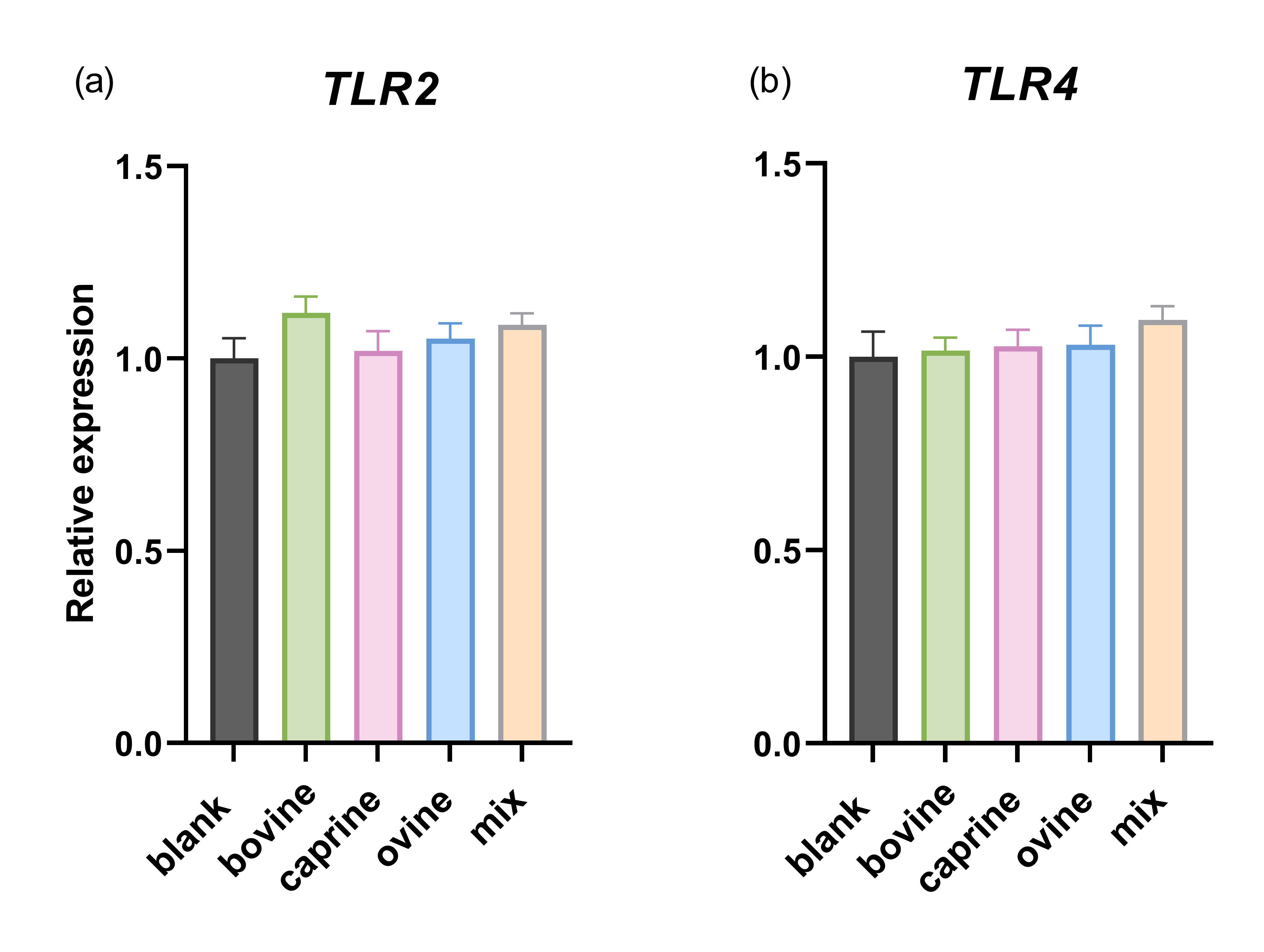

Supplement: Supplementary file 1 [file molecules-30-01261-s001.zip › Figure S2.tif]
